# Malignant potential of oral lichen planus without epithelial dysplasia: a systematic review and meta-analysis of longitudinal studies

**DOI:** 10.3389/fonc.2026.1949729

**Published:** 2026-10-13

**Authors:** Carolina Brás Domingues, João Botelho, António Mano Azul, Pedro Ferreira Trancoso

**Affiliations:** 1 Egas Moniz Center for Interdisciplinary Research, Egas Moniz School of Health & Science, Caparica, Portugal; 2 Oral Surgery and Oral Medicine Department, Egas Moniz School of Health & Science, Caparica, Portugal

**Keywords:** cohort studies, longitudinal, malignant transformation, oral cancer, oral healh, oral lichen planus

## Abstract

**Introduction:**

Oral lichen planus (OLP) is a chronic inflammatory disease of the oral mucosa classified as a potentially malignant oral disease. However, its risk of malignant transformation remains controversial because of heterogeneous diagnostic criteria and methodologies across studies.

**Objective:**

To assess the malignant transformation rate of non-dysplastic OLP through a systematic review and meta-analysis of longitudinal studies.

**Materials and methods:**

A systematic review was conducted according to PRISMA guidelines and registered in PROSPERO (CRD420261295782). PubMed, Embase, and grey literature were searched. Longitudinal observational studies including patients with histopathologically diagnosed OLP, without epithelial dysplasia at diagnosis, and reporting malignant transformation rates were included. The review focused specifically on longitudinal evidence evaluating malignant transformation in patients meeting these eligibility criteria. Methodological quality was assessed using the Newcastle-Ottawa Scale, and meta-analysis was performed using a random-effects model.

**Results:**

Twenty-four studies, comprising 13, 143 patients and 230 cases of malignant transformation, were included. The pooled malignant transformation rate was 1.56% (95% CI: 1.14–2.12%), with substantial heterogeneity (I² = 74.8%). Subgroup analyses showed no significant differences according to study design, geographic region, or diagnostic criteria. Meta-regression identified the male-to-female ratio as a significant moderator, while sample size and follow-up duration had no significant influence. No evidence of publication bias was observed.

**Conclusions:**

The malignant transformation rate of OLP was very low, with a pooled estimate of 1.56%, supporting continued surveillance. Future research should focus on standardized diagnostic approaches and reliable risk stratification models to improve understanding of malignant transformation in OLP and ensure better population representation.

**Systematic review registration:**

https://www.crd.york.ac.uk/PROSPERO/view/CRD420261295782, identifier CRD420261295782.

## 1 Introduction

Oral lichen planus (OLP) is a chronic inflammatory mucocutaneous disease recognized as a potentially malignant condition, characterized by a wide spectrum of clinical manifestations in the oral cavity (1, 2). It is a relatively common chronic inflammatory disease of the oral mucosa, with an estimated prevalence ranging from 1% and 2% in the adult population, although more recent studies suggest overall rates close to or slightly below 1% (3, 4). Although its exact etiology remains unclear, it is widely accepted that it results from a T-cell-mediated immune response directed against basal keratinocytes, triggered by antigenic changes in genetically predisposed individuals (5).

Oral potentially malignant disorders (OPMDs) refer to a group of conditions associated with an increased risk of developing oral squamous cell carcinoma (1). OLP has been included in the group of OPMDs due to documented cases of progression to squamous cell carcinoma, although the magnitude of this risk remains a matter of debate (6). Despite its classification as an OPMD, the magnitude of the malignant transformation risk associated with OLP remains highly controversial. Studies report rates as low as below 0.5%, calling into question the true malignant potential of OLP when strict diagnostic criteria are applied (6). However, more recent systematic reviews point to overall rates in the range of 1% to 1.5% (7, 8).

In addition, dysplastic lesions have long been recognized as a potential source of bias in studies evaluating OLP malignant transformation, since dysplasia itself is a well-established predictor of malignant progression (1). While some authors argue epithelial dysplasia should not be considered an exclusion criterion for the diagnosis of OLP (9), others advocate that lesions presenting epithelial dysplasia should be classified separately as lichenoid lesions (10). Given that epithelial dysplasia is a well-established predictor of malignant progression, its inclusion may introduce an important source of confounding and make it difficult to determine whether malignant transformation is attributable to OLP itself or to pre-existing dysplastic changes. In this regard, exploring malignant transformation by OLP excluding dysplastic lesions would be relevant to provide a more accurate estimate of the intrinsic malignant potential of OLP.

Therefore, the present systematic review and meta-analysis aimed to estimate the malignant transformation risk of non-dysplastic OLP through estimates from longitudinal studies.

## 2 Materials and methods

### 2.1 Study design

This systematic review was conducted according to the methodological recommendations of the Cochrane Handbook (11). A structured protocol was defined *a priori*, and approved by all authors and prospectively registered in PROSPERO (CRD420261295782). Reporting followed the PRISMA 2020 guidelines (12).

### 2.2 Eligibility criteria

#### 2.2.1 Inclusion criteria

Original articles published in English in peer-reviewed journals from 1978 onwards. Longitudinal observational studies (prospective and retrospective cohorts, and case-control studies) including at least 50 individuals diagnosed with OLP, with diagnosis confirmed by histopathological examination and absence of epithelial dysplasia at the time of initial diagnosis.

#### 2.2.2 Exclusion criteria

Case reports, reviews, or articles that did not clearly distinguish between cutaneous and oral forms of lichen planus (LP); cross-sectional studies; cases presenting epithelial dysplasia (including lichenoid dysplasia) at initial diagnosis.

### 2.3 Search strategy

A comprehensive electronic search was performed in PubMed and Embase from database inception until. The search strategy combined Medical Subject Headings (MeSH) and free-text terms:

(“oral lichen planus” OR “oral lichenoid lesion” OR “oral lichenoid reaction” OR “lichen planus”) AND (“malignant transformation” OR “oral cancer” OR “oral squamous cell carcinoma” OR carcinoma OR malignant) AND (“cohort” OR “follow-up” OR longitudinal OR incidence).

Reference lists of included studies and relevant reviews were also manually screened to identify additional eligible studies. No restrictions regarding language or country were initially applied during the search phase.

### 2.4 Study selection

Two independent reviewers (CD and PFT) performed study selection in two stages. Initially, titles and abstracts retrieved from the electronic databases were screened to exclude clearly irrelevant studies. Subsequently, potentially eligible articles underwent full-text assessment. Any disagreement between reviewers was resolved through discussion and consensus with a third reviewer (JB). The reasons for exclusion at the full-text stage were documented and reported in the PRISMA flow diagram. Inter-reviewer agreement was assessed using Cohen’s kappa coefficient.

### 2.5 Data extraction and data items

Two independent reviewers (CD and PFT) extracted data using a standardized extraction form developed *a priori*. The following variables were collected: First author and year of publication; Country and continent; Study design; Recruitment period; Sample size; Follow-up duration; Mean age and sex distribution; Diagnostic criteria used for OLP; Presence or exclusion of epithelial dysplasia at baseline; Clinical subtype of OLP; Anatomical location of lesions; Tobacco and alcohol consumption; Number of malignant transformation events; Risk estimates and associated measures. Whenever additional clarification or missing data were necessary, corresponding authors were contacted by email.

### 2.6 Methodological quality assessment

The methodological quality and risk of bias of included studies were independently assessed by two reviewers using the Newcastle-Ottawa Scale (NOS). Each of the seven domains of the tool (confounding, measurement of exposure, selection of participants, post-exposure interventions, missing data, measurement of outcomes, and selective reporting of results) and the study and outcome overall were rated as having a “low” (between 7 to 9 stars) “moderate” (between 4 to 6 stars) or “high” (between 0–3 stars) risk of bias, according to NOS. The appraisal was conducted by two reviewers independently (CD and JB) in Excel.

### 2.7 Statistical analysis

Extracted data were organized into evidence table (**Supplementary Table 1**). Because malignant transformation represents a proportion-based outcome and due to the expected variability in event rates across studies, pooled estimates were calculated using random-effects meta-analysis with double arcsine transformation, following the methodological approach proposed by Barendregt et al. (13). The random-effects model was selected considering the expected methodological and clinical heterogeneity among studies, including differences in: Diagnostic criteria; Follow-up duration; Study populations; Geographic distribution; Inclusion or exclusion of Oral Lichenoid Lesions (OLL).

Pooled estimates of malignant transformation were reported as proportions with corresponding 95% confidence intervals (95% CI). These proportions were then converted to percentages when presenting and discussing the estimates. Statistical heterogeneity was assessed using Cochran’s Q test; Chi-square test; Higgins’ I. statistic. Substantial heterogeneity was considered when I^2^ exceeded 50%. All analyses were conducted using R software (R Foundation for Statistical Computing, Vienna, Austria), using the “meta” package (14). We also performed sensitivity and subgroup Analyses. *A priori* sensitivity analyses were planned to explore the influence of: Presence or absence of baseline epithelial dysplasia; Risk of bias classification; Follow-up duration.

Subgroup analyses were further performed according to: Clinical subtype (reticular vs erosive/atrophic forms); Anatomical location; Tobacco and alcohol exposure; Geographic region. Meta-regression analyses were planned to evaluate the influence of potential confounding variables on malignant transformation estimates, including mean age; male/female ratio; follow-up duration; geographic latitude; sample size and publication bias.

Publication bias was evaluated using funnel plot inspection and Egger’s regression test whenever 10 publications were included in each pooled estimate (11). A significance level of α = 0.05 was adopted for all statistical analyses.

## 3 Results

### 3.1 Study selection

Of an initial sample of 635 entries, 37 were included for full-text review. In total, 11 studies were excluded for failing to meet the study design criteria, and two study was excluded due to lack of access (**Figure 1**). Inter-rater agreement was excellent, with a coefficient of 0.90 (95% CI: 0.88-0.92).

**Figure 1:**
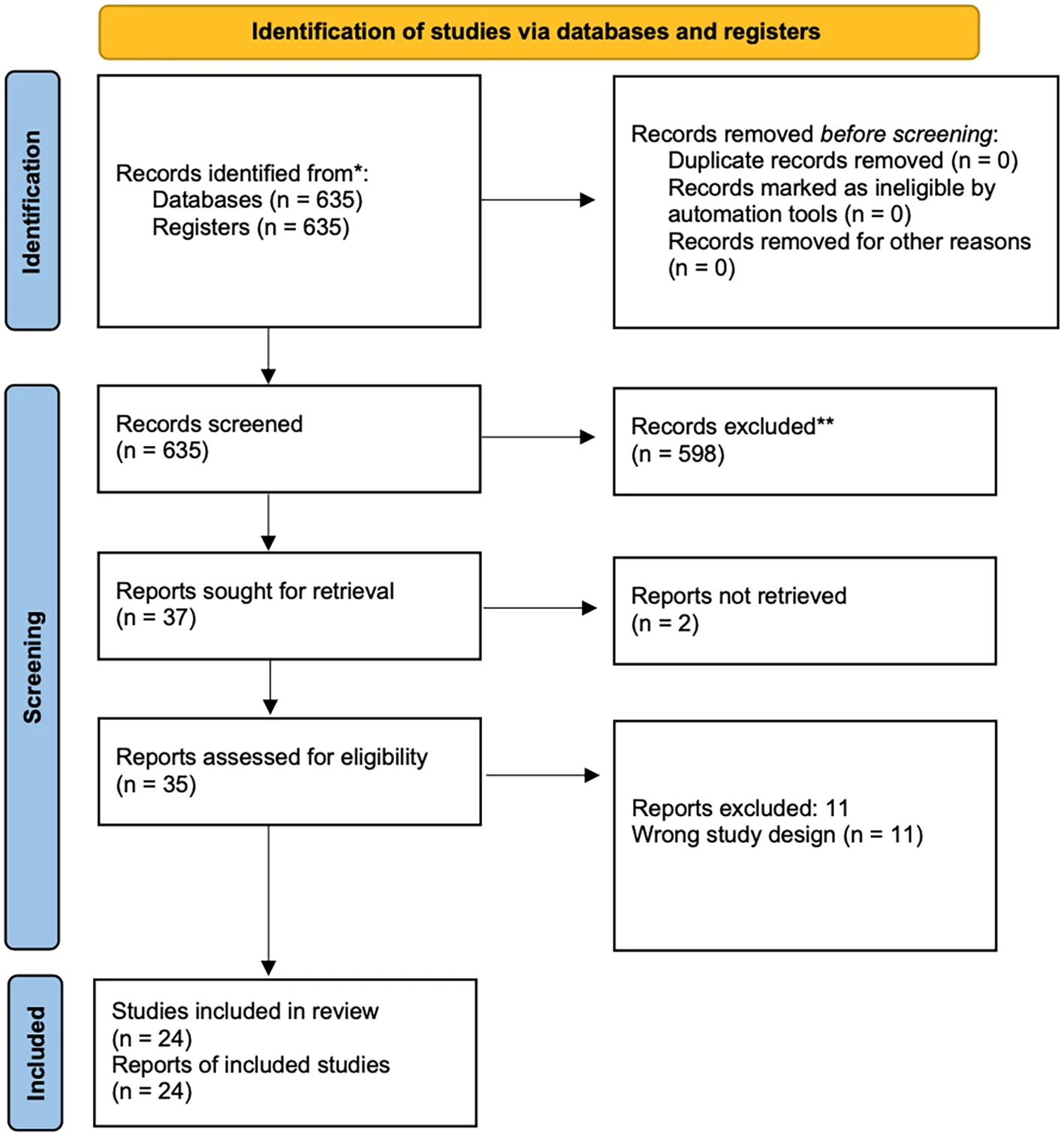
PRISMA 2020 flow diagram illustrating the study selection process for the systematic review and meta-analysis.

### 3.2 Study characteristics

Twenty-four studies published between 1985 and 2026 were included (2, 3, 5, 15–35), comprising 13, 143 patients diagnosed with OLP (**Supplementary Table 1**). Most studies were retrospective cohort studies (66.7%), followed by prospective cohorts (25.0%) and one ambispective (n=1). The majority were conducted in academic settings and originated predominantly from Europe (70.8%), particularly Italy. Sample sizes ranged from 67 to 3, 091 participants. Follow-up periods varied substantially across studies, generally ranging from several years to more than a decade. Diagnostic criteria for OLP differed between studies, including WHO criteria, modified van der Meij and van der Waal criteria, AAOMP recommendations, and combined clinical-histopathological diagnoses. Three studies (2, 431 patients) used the original WHO 1978 criteria, 8 studies (5, 413 patients) used the modified WHO 2003 criteria, and 3 studies (904 patients) used the 2016 AAOMP criteria. One study (565 patients) applied both the modified WHO and AAOMP criteria, whereas the remaining 9 studies (3, 912 patients) used other clinical and/or histopathological diagnostic approaches. This variability may contribute to methodological heterogeneity across the included studies.

### 3.3 Risk of bias in studies

A total of 24 studies were evaluated, with overall scores ranging from 7 to 8 points, corresponding to moderate or low risk of bias. Most studies (18/24; 75.0%) were classified as having a moderate risk of bias (2, 3, 5, 21–35), achieving a total NOS score of 7 points. The remaining six studies (25.0%) were considered to have a low risk of bias, with a score of 8 points (15–20).

Across the NOS domains, there was substantial methodological consistency (detailed information available on **Supplementary File**). All studies adequately ascertained exposure confirmed that the outcome of interest was not present at baseline and considered potential confounding factors through subgroup analyses or multivariable statistical approaches. Likewise, outcome assessment and follow-up duration were judged to be appropriate in all included studies. The main methodological limitations were observed in the selection of the non-exposed cohort and the adequacy of follow-up. All studies presented limitations in the comparability or representativeness of the comparison groups. Furthermore, adequacy of follow-up was the criterion most frequently reducing the overall NOS score. Eighteen studies were rated “d” for this domain, reflecting incomplete follow-up information or potentially important losses during follow-up. In contrast, the six studies classified as low risk of bias received a more favorable rating (“b”) for follow-up adequacy, which contributed to their higher overall scores.

### 3.4 Synthesis of results

A total of 24 studies comprising 13, 143 patients and 230 malignant transformation events were included in the meta-analysis. Using a random-effects model with logit transformation, the pooled malignant transformation rate of OLP was estimated at 1.56% (95% CI: 1.14%-2.12%) (**Figure 2**). Substantial between-study heterogeneity was observed (I^2^ = 74.8%, τ. = 0.425, p < 0.001), indicating considerable variability in malignant transformation rates across studies beyond that expected by chance alone. .

**Figure 2:**
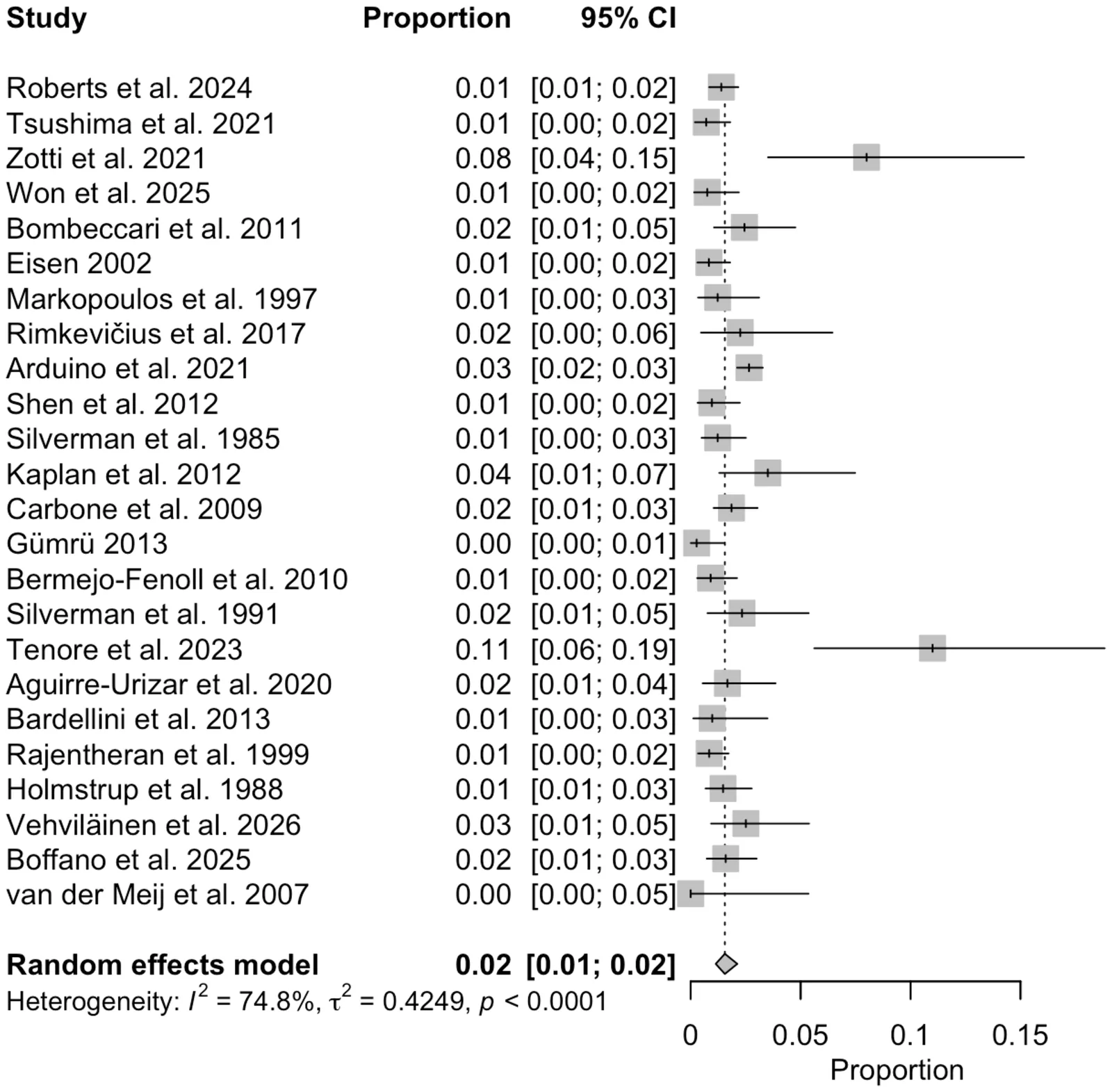
Forest plot of the pooled malignant transformation rate of OLP. The forest plot presents the individual study estimates and corresponding 95% confidence intervals (CIs) for malignant transformation.

Subgroup analyses were performed according to study design, geographic region, and diagnostic classification to explore potential sources of heterogeneity (**Table 1**). No statistically significant differences in malignant transformation rates were observed across any of the evaluated subgroups. However, heterogeneity varied considerably, being higher among retrospective than prospective studies, higher in European and Asian than in North American studies, and higher among studies using case-definition criteria than among those using combined clinical-histopathological diagnostic criteria (**Table 1**). In what study design regards, the pooled malignant transformation rate was similar across retrospective (1.52%, 95% CI: 1.00%-2.32%), prospective (1.66%, 95% CI: 1.18%-2.34%), and ambispective studies (1.67%, 95% CI: 0.70%-3.95%). No significant differences were found between study designs (Q = 0.11, p = 0.947). Heterogeneity was high among retrospective studies (I^2^ = 81.7%) but absent among prospective studies (I^2^ = 0.0%).

**Table 1:** Subgroup analyses of the pooled malignant transformation rate of OLP.

Subgroup	K	Pooled estimate (%)	95% CI (%)	I^2^ (%)	p-value for subgroup difference
Overall	24	1.56	1.14-2.12	74.8	–
Study Design					0.947
Retrospective	17	1.52	1.00-2.32	81.7	
Prospective	6	1.66	1.18-2.34	0.0	
Ambispective	1	1.67	0.70-3.95	NA	
Continent					0.295
Europe	17	1.74	1.18-2.55	77.1	
Asia	3	1.27	0.57-2.78	73.9	
North America	4	1.10	0.72-1.69	19.4	
Diagnostic Classification					0.823
Case definition	15	1.59	0.98-2.59	80.2	
Clinical-histopathological	9	1.49	1.06-2.09	61.0	

K, number of studies; CI, confidence interval; I², percentage of total variability attributable to between-study heterogeneity; NA, not applicable; OLP, oral lichen planus.

Focusing on the geographic region, studies conducted in Europe showed a pooled malignant transformation rate of 1.74% (95% CI: 1.18%-2.55%), compared with 1.27% (95% CI: 0.57%-2.78%) in Asia and 1.10% (95% CI: 0.72%-1.69%) in North America. However, these differences were not statistically significant (Q = 2.44, p = 0.295). Heterogeneity remained substantial in European (I^2^ = 77.1%) and Asian studies (I^2^ = 73.9%), whereas North American studies showed low heterogeneity (I^2^ = 19.4%).

Studies using case-definition criteria reported a pooled malignant transformation rate of 1.59% (95% CI: 0.98%-2.59%), while studies employing combined clinical and histopathological diagnostic criteria reported a rate of 1.49% (95% CI: 1.06-2.09%). No significant subgroup differences were detected (Q = 0.05, p = 0.823). Heterogeneity was higher among studies based on case definitions (I^2^ = 80.2%) than among those using clinical-histopathological confirmation (I^2^ = 61.0%).

### 3.5 Additional analyses

Meta-regression identified the male-to-female ratio as a significant predictor of malignant transformation rates (QM = 4.45, p = 0.035). A positive association was observed between increasing male predominance and the likelihood of malignant transformation (β = 0.756, 95% CI: 0.054-1.458), suggesting that studies with a higher proportion of male participants tended to report higher malignant transformation rates. Despite this finding, substantial residual heterogeneity remained (I^2^ = 73.8%).

In contrast, sample size was not significantly associated with malignant transformation rates (QM = 0.01, p = 0.922). The regression coefficient was close to zero (β < 0.001, 95% CI: −0.0005 to 0.0004), indicating that study size did not influence the reported risk estimates. Similarly, follow-up duration was not a significant moderator of malignant transformation rates (QM = 0.01, p = 0.943). The estimated effect was negligible (β = −0.003, 95% CI: −0.096 to 0.089), suggesting that differences in follow-up length did not explain the observed between-study variability.

Using Egger’s linear regression test, the analysis showed no statistically significant evidence of small-study effects or publication bias (t = −1.99, df = 22, p = 0.059) (**Figure 3**). The findings indicate that publication bias is unlikely to have substantially influenced the pooled estimate of malignant transformation risk in OLP.

**Figure 3:**
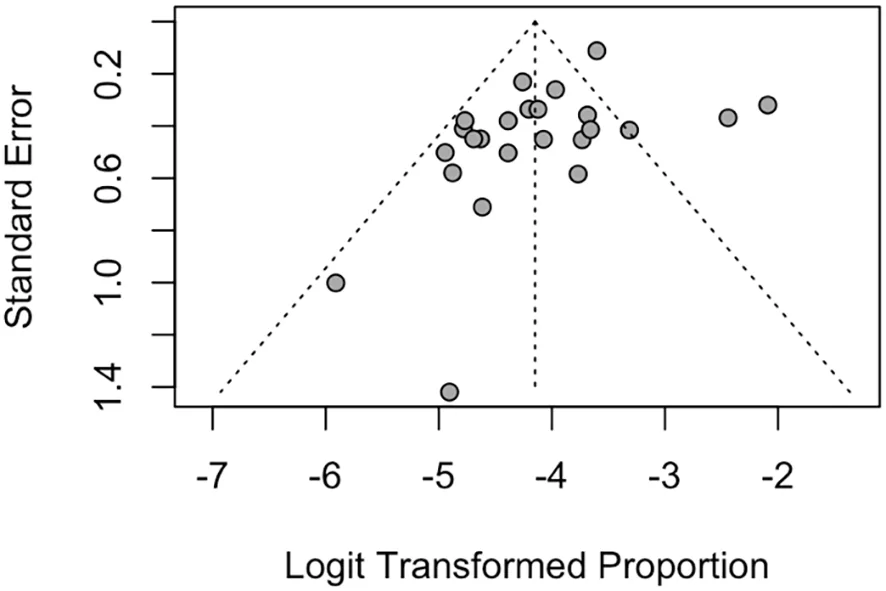
Funnel plot assessing publication bias in studies evaluating the malignant transformation rate of OLP. The funnel plot displays the logit-transformed proportions of malignant transformation against their standard errors.

## 4 Discussion

### 4.1 Summary of main results

The present systematic review and meta-analysis aimed to estimate the malignant transformation rate of OLP. The overall pooled estimate at 1.56% (95% CI: 1.14- 2.12%), supports OLP as a very low risk oral disorder for malignant transformation. In addition, male presented a significant higher risk according to meta-regression values, but no further risk indicators were found due to lack of available data, particularly smoking and alcohol consumption, or chronic medication. Despite the very low risk, the clinical relevance of this finding should not be underestimated, due to the chronic nature of OLP, its prevalence in the general population, and the severe implications of developing oral squamous cell carcinoma.

The chronic and often fluctuating nature of OLP should also be considered when translating these findings into clinical practice. Although the overall risk of malignant transformation appears relatively low, it is not zero. And due to the difficulty to predict whether patients will eventually experience such malignant transformation, regular clinical surveillance and long-term follow-up have been recommended for patients living with OLP (26). This regular follow-up is key in the context of early recognition of such changes to allow timely intervention and potentially improve patient outcomes.

### 4.2 Agreements and disagreements with other reviews or studies

The obtained pooled estimate aligns with previous systematic reviews that reported malignant transformation rates of 1% to 2% (7, 8). Particularly, earlier studies by Aghbari et al. (2017) (7) reported an overall transformation rate of approximately 1.1%, while subsequent reviews by González-Moles et al. (2019) (8).

Nevertheless, the clinical relevance of this finding should also be put in context of the burden of oral cancer. According to the GLOBOCAN 2022 estimates, lip and oral cavity cancer accounted for approximately 389, 846 new cases and 188, 438 deaths worldwide (36, 37). If we apply the obtained malignant rate to a showcase country, assuming an OLP prevalence of 1-2% in Portugal, approximately 100, 000 to 200, 000 individuals may be affected by the disease. If the pooled malignant transformation rate observed in the present review (1.56% and respective 95% CI) were was applied to this estimated population, this would correspond to approximately 1, 560 to 3, 120 individuals potentially developing oral cancer in association with OLP. This estimate appears disproportionate when compared with the 1, 293 new cases of lip and oral cavity cancer reported in Portugal in 2022 by GLOBOCAN (International Agency for Research on Cancer, 2024) (36). One might argue that this example may be overstated but raises the possibility that malignant transformation rates derived from longitudinal clinical cohorts may overestimate the risk when generalized to the overall population. This discrepancy may be partly explained by the inclusion of selected patient populations, referral-center cohorts, and individuals already under specialist follow-up, rather than incident, population-based OLP cases. With this in mind, we suggest that future research should present results concerning newly detected cases within the same inclusion period and respective follow-up.

### 4.3 Evidence quality and potential bias in the review process

Considerable heterogeneity was detected among the studies included (I^2^ = 74.8%). Such variability is expected in such studies, as differences in patient demographics, diagnostic criteria, follow-up procedures, exposure to risk factors, and healthcare environments may substantially influence the reported transformation rates. Similar levels of heterogeneity have also been reported in previous studies (7, 8).

Study design, geographic location, and diagnostic classification were found to unlikely account for the variability reported in the literature. Nevertheless, prospective studies demonstrated substantially lower heterogeneity than retrospective studies, which may reflect more standardized diagnostic procedures, follow-up protocols, and outcome assessment. Additionally, although European studies showed slightly higher transformation rates compared to Asian and North American studies, these differences were not statistically significant.

Another highly debated point in the literature concerns the diagnosis of OLP itself. Over the years, several diagnostic systems have been proposed, including the original WHO criteria (38), the modified criteria proposed by van der Meij and van der Waal (10), and more recently the recommendations of the AAOMP (39). In the present review, no significant differences were observed between studies using case-definition criteria and those employing combined clinical-histopathological diagnostic approaches. Nevertheless, studies based on case definitions demonstrated substantially greater heterogeneity, suggesting that diagnostic inconsistency remains an important methodological limitation (10, 39).

Furthermore, a strength of this review was the exclusion, whenever possible, of lesions presenting epithelial dysplasia at baseline. The inclusion of dysplastic lesions has long been recognized as a potential source of bias in studies evaluating OLP malignant transformation since dysplasia itself is a well-established predictor of malignant progression (1). By focusing on lesions without baseline dysplasia, the present study aimed to provide a more reliable estimate of the malignant potential specifically attributable to OLP. Nevertheless, this issue remains controversial. While González-Moles & Ramos-García (9) have argued that epithelial dysplasia should not be considered an exclusion criterion for the diagnosis of OLP, this position has been challenged by other authors, particularly Van der Meij & Van der Waal (10), who maintain that lesions presenting epithelial dysplasia should be classified separately as lichenoid lesions. Given that epithelial dysplasia is a well-established predictor of malignant progression, its inclusion may introduce an important source of confounding and make it difficult to determine whether malignant transformation is attributable to OLP itself or to pre-existing dysplastic changes. In this regard, the exclusion of dysplastic lesions may provide a more accurate estimate of the intrinsic malignant potential of OLP.

Meta-regression analyses provided additional insights into factors potentially associated with malignant transformation. Among the variables examined, only the male-to-female ratio emerged as a statistically significant moderator. Studies with a higher proportion of male participants tended to report higher rates of malignant transformation. This observation is particularly noteworthy because OLP predominantly affects women (16). Although the underlying explanation remains uncertain, several hypotheses may be considered. Men may present greater exposure to established risk factors for oral squamous cell carcinoma, including tobacco and alcohol consumption (40). Nevertheless, these findings should be interpreted cautiously as meta-regression analyses are based on aggregate study-level data and are therefore susceptible to ecological bias.

The methodological quality of the included studies was overall good. Most studies exhibited a moderate risk of bias, mainly due to limitations related to cohort selection and the adequacy of follow-up, commonly encountered in observational studies with extended follow-up periods. Several studies inadequately reported loss to follow-up and participant drop-out rates, making it difficult to assess the potential impact of attrition bias on the reported results. Consequently, the possibility that some cases were not captured during the observation period cannot be ruled out.

Publication bias was assessed through visual examination of the funnel plot and Egger’s regression test. Although Egger’s test was close to statistical significance (p = 0.059), no definitive evidence of publication bias was found. Nevertheless, the borderline result implies that small-study effects cannot be entirely ruled out. Consequently, the pooled estimate should be interpreted with the understanding that studies reporting very low transformation rates might be underrepresented in the literature.

Several limitations of this review should be acknowledged. First, the substantial heterogeneity observed among studies limits the accuracy of the pooled estimate. Second, key patient-level variables such as the clinical subtype, lesion location, tobacco and alcohol exposure and treatment history were inconsistently reported, hindering more detailed analyses. Third, the inclusion of studies with different diagnostic criteria may have led to some degree of misclassification bias despite efforts to apply strict eligibility criteria. Despite these limitations, this review has notable strengths. It represents one of the largest contemporary syntheses of evidence on OLP malignant transformation, including more than 13, 000 patients from diverse geographic regions. In addition, only longitudinal studies were included, allowing assessment of malignant transformation over time. The exclusion of lesions presenting epithelial dysplasia at baseline further strengthened the validity of the pooled estimate by minimizing a major source of diagnostic and methodological bias. Finally, the use of subgroup analyses, meta-regression, and publication bias assessment provided a comprehensive evaluation of potential sources of heterogeneity and uncertainty.

Future prospective studies with long-term follow-up, ideally extending beyond five years, are needed to improve risk stratification and refine our understanding of malignant transformation in OLP. Attention should be given to the inclusion of representative patient populations, including sex distributions that reflect the epidemiology of the disease, as well as to the adequate control of established risk factors for oral cancer, such as tobacco and alcohol consumption. The adoption of standardized diagnostic criteria and detailed clinicopathological characterization of patients and lesions will also be essential to enhance the quality and comparability of future evidence.

## 5 Conclusions

The overall pooled estimate of 1.56% indicates that OLP has a very low malignant transformation. Future studies are required with standardized diagnostic approaches and reliable risk stratification models with a better population representation within studies.

## Data Availability

The original contributions presented in the study are included in the article/**Supplementary Material**. Further inquiries can be directed to the corresponding author.
